# Supplementary material for: Peak Power: A Severity Measure for Head Acceleration Events Associated with Suspected Concussions
Source: Sports Med. 2025 Sep 19;56(2):589–97. doi: 10.1007/s40279-025-02308-0 (PMC12982200; doi:10.1007/s40279-025-02308-0)

Supplementary Material 1. Paired-sample area under the curve (AUC) comparisons based on the receiver operating characteristic analysis in the men’s and women’s games. Significant differences are represented by an asterisk (*).

| Test Pairs | AUC Difference | 95% CI  Lower Bound | 95% CI  Upper Bound | p-value |
| --- | --- | --- | --- | --- |
| **Men** |  |  |  |  |
| Power - MPS | 0.012 | -0.007 | 0.032 | 0.214 |
| Power - HARM | 0.006 | -0.017 | 0.030 | 0.594 |
| Power - PAA | 0.024 | -0.011 | 0.059 | 0.179 |
| Power - PLA | 0.013 | 0.000 | 0.027 | 0.052 |
| Power - dPAV | 0.034 | 0.006 | 0.061 | 0.016* |
| MPS - HARM | -0.006 | -0.015 | 0.003 | 0.167 |
| MPS - PAA | 0.012 | -0.017 | 0.040 | 0.418 |
| MPS - PLA | 0.001 | -0.022 | 0.024 | 0.940 |
| MPS - dPAV | 0.021 | 0.007 | 0.035 | 0.003* |
| HARM - PAA | 0.018 | -0.006 | 0.042 | 0.147 |
| HARM - PLA | 0.007 | -0.018 | 0.032 | 0.581 |
| HARM - dPAV | 0.027 | 0.013 | 0.041 | 0.001* |
| **Women** |  |  |  |  |
| Power - MPS | 0.074 | -0.007 | 0.154 | 0.074 |
| Power - HARM | 0.040 | -0.023 | 0.102 | 0.215 |
| Power - PAA | 0.006 | -0.078 | 0.089 | 0.892 |
| Power - PLA | -0.024 | -0.064 | 0.016 | 0.241 |
| Power - dPAV | 0.102 | 0.038 | 0.165 | 0.002* |
| MPS - HARM | -0.034 | -0.078 | 0.011 | 0.135 |
| MPS - PAA | -0.068 | -0.108 | -0.028 | 0.001* |
| MPS - PLA | -0.098 | -0.154 | -0.041 | 0.001* |
| MPS - dPAV | 0.028 | -0.027 | 0.084 | 0.316 |
| HARM - PAA | -0.034 | -0.076 | 0.008 | 0.112 |
| HARM - PLA | -0.064 | -0.110 | -0.018 | 0.006* |
| HARM - dPAV | 0.062 | 0.026 | 0.098 | 0.001* |

Supplementary Material 2. Precision recall curves for the men’s and women’s game with area under the precision recall curves (AUPRC).

**Men**


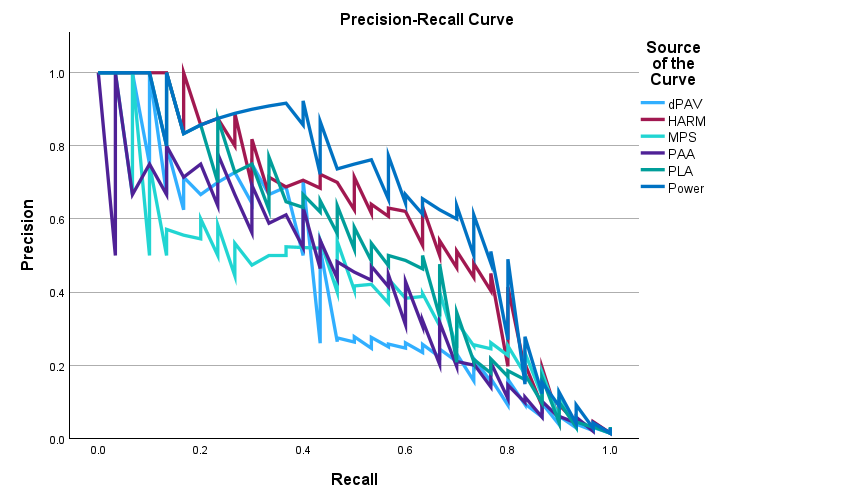


**Women**


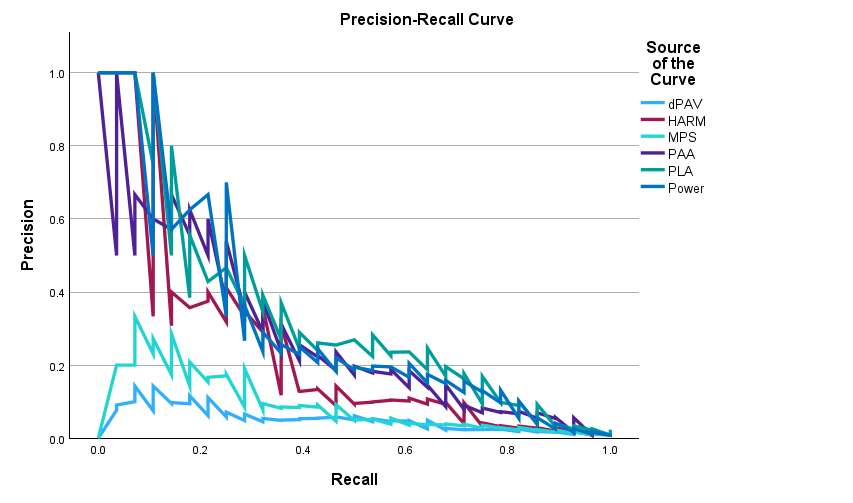

Supplement: Supplementary file 1 — Supplementary file1 (DOCX 236 KB) [file 40279_2025_2308_MOESM1_ESM.docx]
